# Supplementary material for: Proteotranscriptomic Profiling of the Toxic Mucus of Kulikovia alborostrata (Pilidiophora, Nemertea)
Source: Toxins (Basel). 2024 Dec 26;17(1):5. doi: 10.3390/toxins17010005 (PMC11768458; doi:10.3390/toxins17010005)
Supplement: Supplementary file 1 [file toxins-17-00005-s001.zip › Supplementary File S4.zip!/ORF 006024 LINALB/230688690e3e92c6/dom_summary.html]

Phyre 2 Results for ORF\_006024\_LINALB

|  |  |
| --- | --- |
| Email | vas9i-kz@mail.ru |
| Description | ORF\_006024\_LINALB |
| Date | Fri Apr 7 01:53:58 BST 2023 || Unique Job ID | 230688690e3e92c6 |

|  |  |
| --- | --- |
| Domain analysis |  |

| Rank | Aligned region |
| --- | --- |

|  |  |
| --- | --- |
| 1 | --- \| c6av8A\_ \| --- |
| 2 | --- \| c7rz3A\_ \| --- |
| 3 | --- \| c5t4rA\_ \| --- |
| 4 | --- \| c5we3A\_ \| --- |
| 5 | --- \| d1qk7a\_ \| --- |
| 6 | --- \| c6f61A\_ \| --- |
| 7 | --- \| c7skcA\_ \| --- |
| 8 | --- \| c1s6xA\_ \| --- |
| 9 | --- \| c1qfdA\_ \| --- |
| 10 | --- \| d1clvi\_ \| --- |
| 11 | --- \| c8feyA\_ \| --- |
| 12 | --- \| d1en2a2 \| --- |
| 13 | --- \| d1ehda2 \| --- |
| 14 | --- \| c7namB\_ \| --- |
| 15 | --- \| c6f8eA\_ \| --- |
| 16 | --- \| c2n86A\_ \| --- |
| 17 | --- \| c1zwuA\_ \| --- |
| 18 | --- \| c2m9lA\_ \| --- |
| 19 | --- \| c2a2vA\_ \| --- |
| 20 | --- \| c2jtbA\_ \| --- |
| 21 | --- \| --- \| --- |
| 22 | --- \| --- \| --- |
| 23 | --- \| --- \| --- |
| 24 | --- \| --- \| --- |
| 25 | --- \| --- \| --- |
| 26 | --- \| --- \| --- |
| 27 | --- \| --- \| --- |
| 28 | --- \| --- \| --- |
| 29 | --- \| --- \| --- |
| 30 | --- \| --- \| --- |
| 31 | --- \| --- \| --- |
| 32 | --- \| --- \| --- |
| 33 | --- \| --- \| --- |
| 34 | --- \| --- \| --- |
| 35 | --- \| --- \| --- |
| 36 | --- \| --- \| --- |
| 37 | --- \| --- \| --- |
| 38 | --- \| --- \| --- |
| 39 | --- \| --- \| --- |
| 40 | --- \| --- \| --- |
| 41 | --- \| --- \| --- |
| 42 | --- \| --- \| --- |
| 43 | --- \| --- \| --- |
| 44 | --- \| --- \| --- |
| 45 | --- \| --- \| --- |
| 46 | --- \| --- \| --- |
| 47 | --- \| --- \| --- |
| 48 | --- \| --- \| --- |
| 49 | --- \| --- \| --- |
| 50 | --- \| --- \| --- |
| 51 | --- \| --- \| --- |
| 52 | --- \| --- \| --- |
| 53 | --- \| --- \| --- |
| 54 | --- \| --- \| --- |
| 55 | --- \| --- \| --- |
| 56 | --- \| --- \| --- |
| 57 | --- \| --- \| --- |
| 58 | --- \| --- \| --- |
| 59 | --- \| --- \| --- |
| 60 | --- \| --- \| --- |
| 61 | --- \| --- \| --- |
| 62 | --- \| --- \| --- |
| 63 | --- \| --- \| --- |
| 64 | --- \| --- \| --- |
| 65 | --- \| --- \| --- |
| 66 | --- \| --- \| --- |
| 67 | --- \| --- \| --- |
| 68 | --- \| --- \| --- |
| 69 | --- \| --- \| --- |
| 70 | --- \| --- \| --- |
| 71 | --- \| --- \| --- |
| 72 | --- \| --- \| --- |
| 73 | --- \| --- \| --- |
| 74 | --- \| --- \| --- |
| 75 | --- \| --- \| --- |
| 76 | --- \| --- \| --- |
| 77 | --- \| --- \| --- |
| 78 | --- \| --- \| --- |
| 79 | --- \| --- \| --- |
| 80 | --- \| --- \| --- |
| 81 | --- \| --- \| --- |
| 82 | --- \| --- \| --- |
| 83 | --- \| --- \| --- |
| 84 | --- \| --- \| --- |
| 85 | --- \| --- \| --- |
| 86 | --- \| --- \| --- |
| 87 | --- \| --- \| --- |
| 88 | --- \| --- \| --- |
| 89 | --- \| --- \| --- |
| 90 | --- \| --- \| --- |
| 91 | --- \| --- \| --- |
| 92 | --- \| --- \| --- |
| 93 | --- \| --- \| --- |
| 94 | --- \| --- \| --- |
| 95 | --- \| --- \| --- |
| 96 | --- \| --- \| --- |
| 97 | --- \| --- \| --- |
| 98 | --- \| --- \| --- |
| 99 | --- \| --- \| --- |
